# Supplementary figures and images for: Mental health stigma and professional help-seeking attitudes a comparison between Cuba and Germany
Source: PLoS One. 2021 Feb 11;16(2):e0246501. doi: 10.1371/journal.pone.0246501 (PMC7877775; doi:10.1371/journal.pone.0246501)

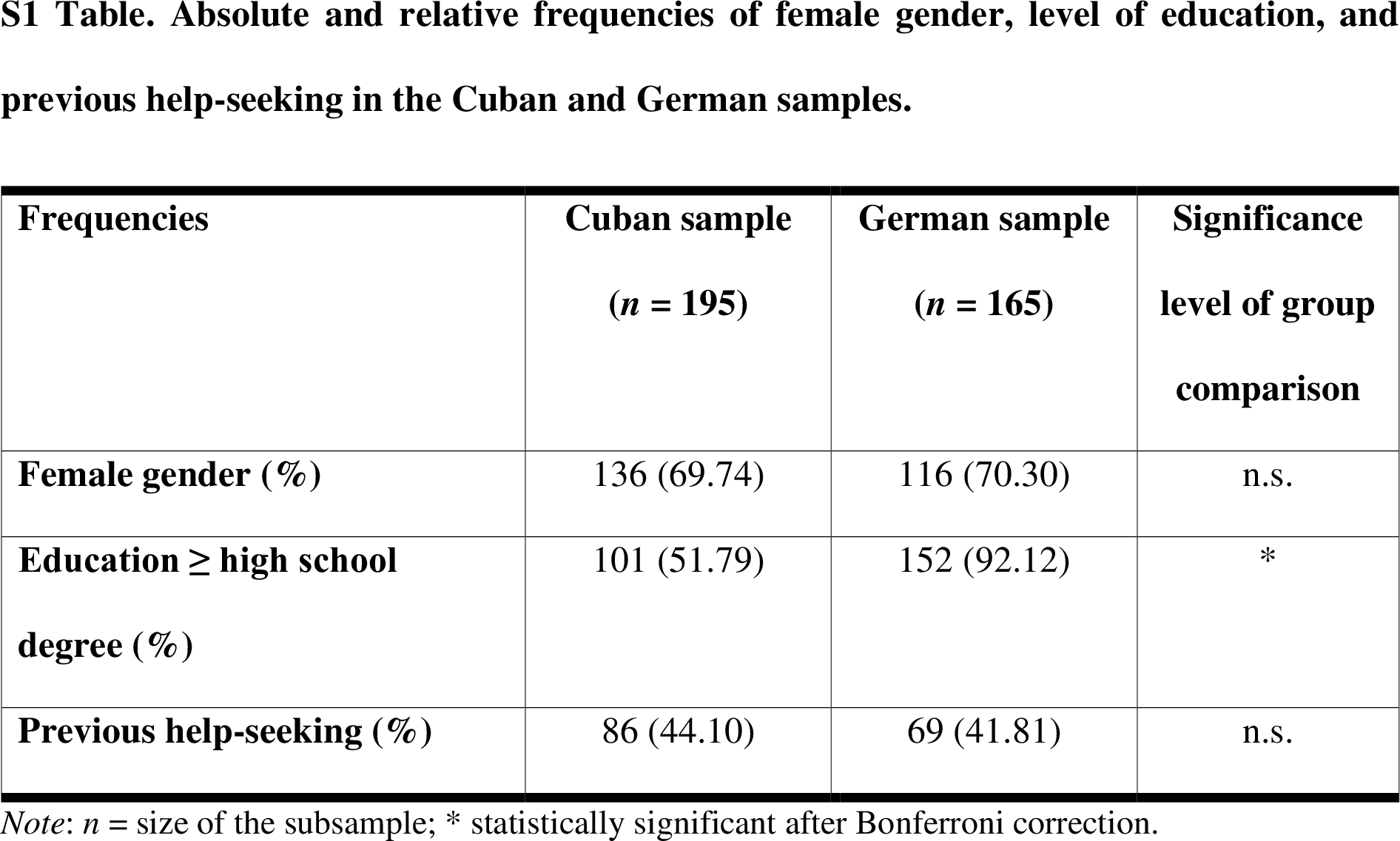

Supplement: S1 Table — n = size of the subsample; * statistically significant after Bonferroni correction (p <.005). (TIF) [file pone.0246501.s001.tif]

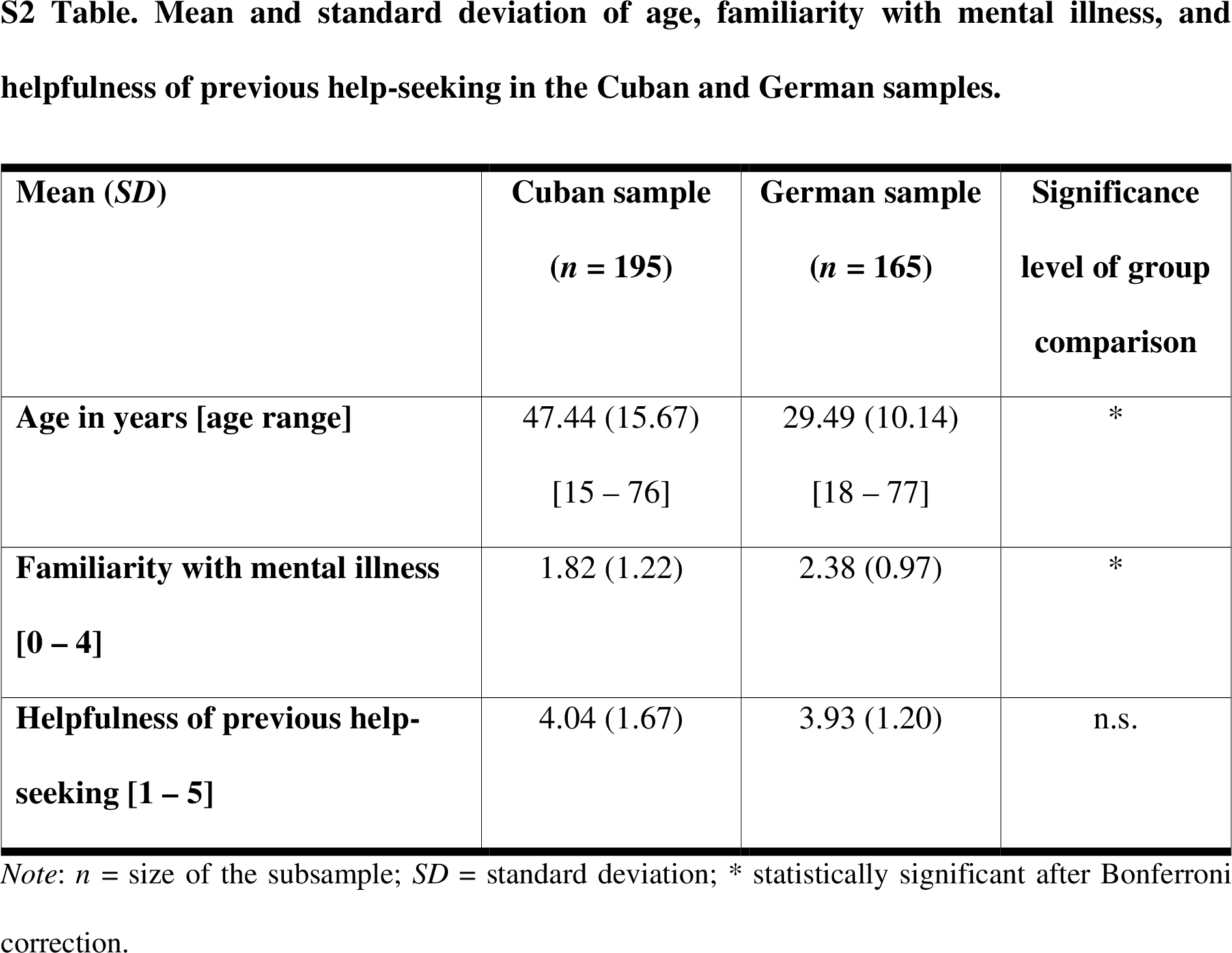

Supplement: S2 Table — n = size of the subsample; SD = standard deviation; * statistically significant after Bonferroni correction (p <.005). (TIF) [file pone.0246501.s002.tif]

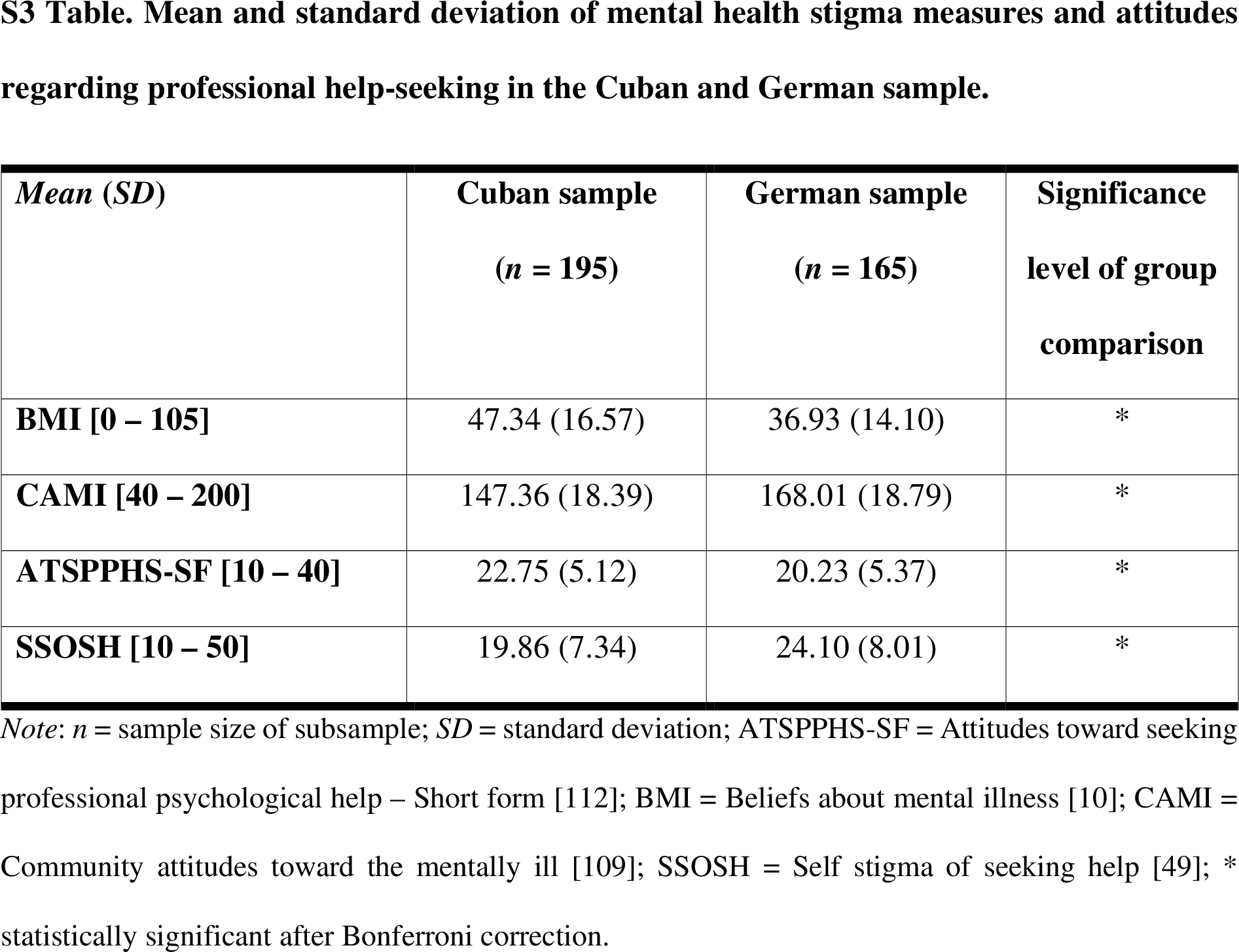

Supplement: S3 Table — n = sample size of subsample; SD = standard deviation; ATSPPHS-SF = Attitudes toward seeking professional psychological help—Short form [112]; BMI = Beliefs about mental illness [10]; CAMI = Community attitudes toward the mentally ill [109]; SSOSH = Self-stigma of seeking help [49]; * statistically significant after Bonferroni correction (p <.005). (TIF) [file pone.0246501.s003.tif]
