# Supplementary material for: Mental health stigma and professional help-seeking attitudes a comparison between Cuba and Germany
Source: PLoS One. 2021 Feb 11;16(2):e0246501. doi: 10.1371/journal.pone.0246501 (PMC7877775; doi:10.1371/journal.pone.0246501)
Supplement: Attachment — Submitted filename: PLOS ONE REVIEW.docx [file pone.0246501.s004.docx]

Manuscript Number: PONE-D-20-28889

Article Type: Research Article

Full Title: Mental health stigma and professional help-seeking attitudes - A comparison between

Cuba and Germany

Short Title: Mental health stigma and help-seeking attitudes

Corresponding Author: Ulrike Buhlmann

Westfalische Wilhelms-Universitat Munster

Muenster, GERMANY

Keywords: Beliefs about mental illness, community attitudes, familiarity with mental illness, gender

Roles

COMMENTS.

This is a well written manuscript that focuses on the important subject matter of mental health stigma.

1. There is a need for a bit more description of the sampling method. It clearly is a sample of convenience/ purposive sample. Kindly give a bit more description of regions it surveyed and how the survey was spread in thes 2 settings. I am however glad that this is highlighted as a limitation at the end of the manuscript.
2. The important aspect of ethical considerations was however left out in the manuscript proper. I would like the authors to include a paragraph on ethical considerations just before the results section. Issues on Ethical approval, consent/autonomy, confidentiality, anonymity, beneficience/maleficience should all be duely highlighted as needed.

The manuscript may be considered for publication by editor once these have been done

Thank you
